# Health-related quality of life (EQ-5D + C) among people living in artisanal and small-scale gold mining areas in Zimbabwe: a cross-sectional study

**DOI:** 10.1186/s12955-020-01530-w

**Published:** 2020-08-18

**Authors:** Friederike-Marie Butscher, Stefan Rakete, Myriam Tobollik, Viola Mambrey, Dingani Moyo, Dennis Shoko, Shamiso Muteti-Fana, Nadine Steckling-Muschack, Stephan Bose-O’Reilly

**Affiliations:** 1 Institute for Medical Information Processing, Biometry and Epidemiology - IBE, LMU Munich, Munich, Germany; 2 Pettenkofer School of Public Health, Munich, Germany; 3 Department of Sport and Health Sciences, Technical University of Munich, Georg-Brauchle-Ring 62, 80992 Munich, Germany; 4 Institute and Clinic for Occupational, Social and Environmental Medicine, LMU University Hospital Munich, Ziemssenstr. 1, D-80336 Munich, Germany; 5 Department Environment and Health, School of Public Health, Bielefeld University, Bielefeld, Germany; 6 Section exposure assessment and health indicators, German Environment Agency, Berlin, Germany; 7 University of the Witwatersrand, School of Public Health, Faculty of Health Sciences, Occupational Health Division, 7 York Road, Parktown, Johannesburg, RSA; 8 Midlands State University, Faculty of Medicine & Faculty of Social Sciences, P Bag, 9055 Gweru, Zimbabwe; 9 Tailjet Consultancy Services, Harare, Zimbabwe; 10 Department of Community Medicine, UZ College of Health Sciences, Harare, Zimbabwe; 11 Institute of Public Health, Medical Decision Making and Health Technology Assessment, UMIT Private University for Health Sciences, Medical Informatics and Technology, Eduard Wallnoefer Center I, 6060 Hall in Tirol, Austria

**Keywords:** Health-related quality of life (HRQoL), EuroQol (EQ), Visual analogue scale, Artisanal and small-scale gold mining (ASGM), Mercury, Mercury intoxication, Zimbabwe

## Abstract

**Background:**

In Zimbabwe, an estimated 500,000 people work in the sector of artisanal and small-scale gold mining (ASGM). Two million Zimbabweans are dependent on this sector. Using mercury is common to extract gold from ore. Long term exposure to mercury can cause various adverse health conditions including chronic mercury intoxication. The influence of these adverse health effects on the health-related quality of life (HRQoL) is still unknown. The aim of this study is to assess the HRQoL of people who identify themselves as miners, and to analyze potential influencing factors, such as age, years of working with mercury and health conditions caused by mercury exposure.

**Methods:**

This cross-sectional study assessed the HRQoL using the standardized EQ-5D + C (3 L) questionnaire and collected human specimens (blood, urine) of people living and possibly working in ASGM areas in Zimbabwe. Factors such as age, years of working with mercury and adverse health conditions possibly caused by mercury exposure were analyzed with regards to their influence on the HRQoL.

**Results:**

The 207 participants (82% male, mean age 38 years) reported 40 different health states. Of the study participants 42.5% reported to be in complete good health while 57.5% reported being unwell in different ways. Nine participants (4.3%) were identified with chronic mercury intoxication, whereas 92 participants (33.3%) had mercury levels above the “Alert” threshold in at least one specimen. Having chronic mercury intoxication has a significant negative influence on the HRQoL, when taking into account age, gender and years of working with mercury. Cognitive problems were the most reported in the questionnaire, however, the association between this domain separately and the HRQoL was not verified.

**Conclusion:**

This study shows that adverse health effects caused by chronic exposure to mercury, have a negative influence on the HRQoL among people living in ASGM areas.

## Introduction

In areas of artisanal and small-scale gold mining (ASGM), people are exposed to several health risks, including mercury exposure [1, 2]. Mercury is a highly toxic metal and negatively affects human health [3]. Mercury causes damage to the central and peripheral nervous system. It can also affect the immune system, lungs and kidneys [3]. Tremors, insomnia, memory loss, neuromuscular effects, headaches and cognitive and motor dysfunction can be observed after inhalation, ingestion or dermal application [4]. A long-term exposure to mercury can lead to chronic mercury intoxication (CMI) [1, 5, 6]. Industrial processes can bring mercury to be released into the air, water and soil whereby it is emitted into the environment [7]. The ‘Minamata Convention on Mercury’ is an agreement of currently 128 governments [8] with the aim “to protect human health and the environment from anthropogenic emissions and releases of mercury” [9]. “Each Party that has artisanal and small-scale gold mining […] within its territory shall take steps to reduce, and where feasible eliminate, the use of mercury” [9].

The largest source of anthropogenic mercury emission is ASGM with a proportion of 37% of world mercury emissions [10]. Among the different methods in ASGM to extract the gold from the ore [1], using mercury is most common one [1, 10] because it is easily applicable and cheap [11]. The gold mining process consists of several steps, beginning with the extraction and processing of the ore. During panning the crushed ore is swiveled with mercury for amalgamation. The amalgam consisting of gold and mercury is then burnt in order to recover the gold, while the mercury vaporizes causing exposure to miners and surrounding people [1]. In addition, the released mercury can get into water and fish and contaminate the human food-chain [11].

The amount of mercury released is directly connected to the technology used [12]. In African countries the level of technology for ASGM processes is lowest and least efficient [12]. In Zimbabwe an estimated 500,000 people work in ASGM [12, 13] and a total of about two million Zimbabweans are dependent on the sector of ASGM [14]. While there has been an increase in gold production in Zimbabwe, there has also been an increase in ASGM activities [14]. Findings indicate that Zimbabwe suffers from some of the highest levels of mercury pollution [14].

Globally, an estimated 14–19 million people work in the sector of ASGM [12, 15]. Often ASGM is informal and poverty driven [16, 17]. Mercury exposure in ASGM is a major and global problem, but often it is neglected [2]. It is therefore essential to be aware of the adverse health effects as well as the health-related quality of life (HRQoL) of people living in ASGM areas to promote and support the implementation of the Minamata Convention [18]. HRQoL is employed to measure the impact of diseases and adverse health effects on the every-day life and thus serves as an important indicator for a holistic understanding [19, 20] of the challenges faced by people living in ASGM area.

Prior studies assessed the quality of life among miners in ASGM in Ghana [18]. Furthermore, the HRQoL among miners in ASGM in Zimbabwe has been investigated and compared with Zimbabwean urban population [21]. The health states, as part of the HRQoL, for a moderate and a severe form of chronic metallic mercury vapor intoxication were assessed using expert interviews [22].

The self-reported HRQoL of individuals having a mercury caused health conditions is still unknown. Furthermore, it is still unknown which factors influence the HRQoL.

### Aim of this study

The aim of this study is to assess the HRQoL of people who identify themselves as miners, in the following described as people living in ASGM areas in Zimbabwe and to analyze potential influencing factors, such as age, years of working with mercury and health conditions caused by mercury exposure. This is a cross-sectional study with a convenience sample of adults living in two districts of Zimbabwe. Health-related quality of life was evaluated using the “Experimental Version Health Questionnaire EQ-5D+C (3L)”. We assume that adverse health conditions have a negative influence on the HRQoL as well as a long working duration with mercury.

Therefore, the two research questions are as follows:

- How is the HRQoL of people living in ASGM areas in Zimbabwe?
- Which factors influence the HRQoL of people living in ASGM areas in Zimbabwe?

## Methods

### Data collection

In this cross-sectional study, data from 207 participants were collected during a field study in March 2019 in two districts of Zimbabwe, Kadoma and Shurugwi. Most gold produced from ASGM comes from the three districts Kadoma, Kwekwe and Shurugwi [23]. Kadoma and Shurugwi are old mining towns [24]. Kadoma District is in Mashonaland West province, has a population of approx. 90.000 people [25] and the highest density of miners in Zimbabwe [24]. Shurugwi is in Midlands Province, population of approx. 20.000 people [25]. These two districts are located approximately 150 and 300 km west and southwest from the capital, Harare, in both areas Shona is the dominant language [26].

The protocol was approved by the ethics committee of the Medical Research Council and the Research Council of Zimbabwe (MRCZ/A/2367, September 26, 2018 and February 25, 2019) and of the Ludwig Maximilians University of Munich, Germany (18–421, October 15, 2018).

The field study lasted 2 weeks and recruitment was conducted on-site using the snowball system. For this sampling technique participants recruited further participants among their colleagues. Local project partners planned and enabled access to people living in ASGM areas in Zimbabwe, the target population. They promoted the study and organized transport from the workplace to the study sites for the participants.

The minimum age of participation was 18 years. All females and males that identified themselves as miners and worked for at least 1 month as miners were included. Only participants who gave their informed consent were included in the study. It was ensured that participants understood the patient information and consent forms, which were available in English, Shona and Ndebele. Participants with low literacy were supported by local staff who spoke the native languages. A medical doctor and two nurses conducted a standardized questionnaire and a medical examination at Kadoma and Shurugwi district hospitals. The team received a one-day training on how to conduct the examination and were supervised and supported during the first examinations. Human specimens such as blood and urine were collected. To avoid a contamination of the human specimens, the data collection was conducted in health centers and not at the mining site. In order to compensate for their loss of income on the study day, the participants received 5 US dollars for participation.

### Questionnaire

The questionnaire consisted of three parts. It was in line with the recommendations by the United Nations Environmental Program (UNEP) on how to identify populations at risk for mercury exposure [27].

Demographic data such as gender (‘male’, ‘female’) and age (in years) was collected. Answers to questions concerning work, such as type (‘smelting amalgam to recover the gold’, ‘no smelting but extracting gold from the ore with mercury’, ‘gold buyer/ smelting gold’, ‘any other or no job’), duration (years working with mercury) and the frequency of alcohol consumption (‘never’, ‘at least once a month’, ‘at least once a week’, ‘at least once a day’) was recorded. Participants were also asked if they are “healthy now” (‘yes’,‘no’), and if they denied, they were asked why. This part of the questionnaire was available in English only, answered verbally by the participants and filled in by the study nurses.

The second part assessed the medical score sum (MSS), which is a toolkit developed by Doering et al. [28] based on Drasch et al. [29]. The ten most significant symptoms defining CMI were identified in the analysis of the health situation of gold miners. This ten-item toolkit assessed six objective tests (ataxia of gait, bluish discoloration of gums, dysdiadochokinesia, heel-to-shin ataxia, match box test for intentional tremor and concentration, pencil tapping test for intentional tremor and coordination), three self-reported items (excessive salivation, sleep disturbances, perceived tremor) and proteinuria (tested with commercial reagent strips for urinalysis) [29]. Each item was coded binary, accordingly adding one score point in case of a positive test result or a present symptom, resulting in a MSS between 0 and 10 for each participant. This part of the questionnaire was available in English only and was filled in by a specially trained doctor, who carried out the examination.

In the last part of the questionnaire, the HRQoL was assessed using the EQ-5D + C (3 L) questionnaire. The EQ-5D was developed by the 1987 established EuroQuol group to provide a generic measure of health [30]. It is updated continuously and there are three versions [31]. For this study the “Experimental Version Health Questionnaire EQ-5D+C (3L)” was provided and approved by the EuroQol (EQ) association. This standardized and cognitively undemanding tool, which can be completed in a short amount of time and consists of two parts. The first part consists of five dimensions (5D) with the additional dimension cognition (+C), that are rated with three levels (3 L). The second part is the visual analogue scale ranging from 0 to 100.

The five dimensions (5D) are: mobility, self-care, usual activities, pain/ discomfort and anxiety/ depression. For each dimension three levels (3 L) can be chosen: no problems (1), some problems (2) or extreme problems (3) [31]. A sixth dimension cognition (+C) was added to the questionnaire as it was done previously [32, 33]. A prior study showed that problems in the cognitive dimension were often reported among gold miners in Zimbabwe [21]. Using the EQ-5D + C, perceived health states of the participants can be expressed by a code reflecting the level of problem for each dimension (‘111111’ represents a health state with no problem in any dimension, ‘333333’ represents a health state with extreme problems in every dimension) [31]. The positions of the ratings represent the sequence of dimensions mentioned above, with mobility on first and cognition on the sixths position.

In the second part of the EQ-5D + C (3 L), participants state their perceived health on visual analogue scale (VAS), which is a scale from 0 ‘worst health you can imagine’ to 100 ‘best health you can imagine’, the higher the score the higher is the self-reported health. Participants are asked to state their ‘overall health today’ by marking a value on the scale [31]. Besides the English version, a version in Shona was also provided, which has been validated beforehand [32]. Participants with low literacy or knowledge of Ndebele only, were supported by the study nurses.

### Human specimens

In addition to the questionnaire, human specimens were collected in order to measure the mercury level in the blood and urine of the participants. To measure inorganic mercury levels spot urine samples were analyzed on site on the same day of collection. The analysis was performed using cold vapour atom absorption spectrometry method (CV-AAS) with a Lumex® mobile mercury analyser (RA-915+) with a RP-91 liquid attachment (Ohio Lumex Co., Solon, OH, USA), (Lumex Analyser RA-951+). The detection level is 0.5 μg/l. All values below 0.5 μg/l were set on 0.25 μg/l, half of the detection level [34]. Blood samples were transported by constant storage at 4 °C to the laboratory of the Institute and Outpatient Clinic for Occupational, Environmental and Social Medicine University Hospital, Germany, stored at − 18 °C and analyzed using DMA80-evo instrument (MLS-Mikrowellen, Leutkirch, Germany).

### Outcome variables

The data collection yielded several variables. The EQ-5D + C (3 L) provides six health dimensions with three levels, the health states. The VAS is available as a metric variable with values between 0 and 100.

In order to use the health states as outcome in the regression analysis they were converted into health utilities (HU). The HU represent the EQ-5D + C (3 L) health states as a single summary number (also known as index values or preference weights). In 2003 a value set for Zimbabwe was assessed, in which a Zimbabwean population sample was asked to value EQ-5D health states [35]. Deriving from those values, weights for each level in each dimension can be calculated by deducting the appropriate weights from 1, the value for full health (‘11111’) [31]. Therefore, every possible health state can be weighted and calculated into a single summary number between 0 and 1, the HU [31]. This number reflects how good or bad a health state is based on the preferences of the general population of Zimbabwe [31]. The HU were multiplied with 100, therefore the VAS and HU values are between 0 and 100.

The outcomes are the values of the VAS and the HU.

### Exposure variables

The exposures of interest are adverse health conditions caused by chronic mercury exposure. Variables were assessed that give information about a health conditions caused by chronic mercury exposure, the mercury levels in human specimens and the MSS. On basis of this information the diagnosis CMI based on the diagnostic tool by Doering et al. [28] was done.

The mercury levels of urine and blood were classified based on the Human Biomonitoring (HBM) values [36] in three categories, respectively. The exposure limit values for each specimen are shown in Table 1. The exposure of mercury in blood and urine were combined into one variable with the categories ‘Mercury in both specimens below 1st exposure limit value’, ‘Mercury at least in one specimen above 1st exposure limit value’ and ‘Mercury at least in one specimen above 2nd exposure limit value’ (Table 2). The MSS was available as a metric score between 0 and 10, the higher the score, the more symptoms a participant showed or reported. For assessing the CMI, the MSS was cut into three categories (Table 2). Not every of the ten components of the MSS were available for all participants. If the MSS category was clearly identifiable despite the missing value, the participant was added to the corresponding category. If the MSS category was not clearly identifiable, the participants value remained ‘missing’.

**Table 1:** Exposure limit values of mercury in urine and blood

	HBM urine (μg/l)	HBM blood (μg/l)	
below 1st HBM exposure limit value	<= 7	<=5	Low level
1st to 2nd HBM exposure limit value (HBM I)	> 7 to <= 25	> 5 to <= 15	Alert level
Over 2nd HBM exposure limit value (HBM II)	> 25	> 15	High level

Table adapted from [31], *HBM* Human Biomonitoring

**Table 2:** Assessment of chronic mercury intoxication (CMI)

	MSS 0–2	MSS 3–4	MSS 5–10
HBM combined			
Mercury in both specimens below HBM I	–	–	–
Mercury at least in one specimen between HBM I and HBM II	–	–	+
Mercury at least in one specimen above HBM II	–	+	+

− = no chronic mercury intoxication, + = chronic mercury intoxication,

*HBM I* 1st exposure limit value, *HBM II* 2nd exposure limit value, *MSS* Medical score sum

For the status of CMI, the three categories of mercury exposure in the specimens were combined with the three categories of MSS values. Participants were classified as chronic mercury intoxicated with a MSS of 3 or more and a mercury exposure above the 2nd exposure limit value in least one specimen or with a MSS of 5 or more and a mercury exposure at least in one specimen above the 1st exposure limit value as shown in Table 2.

For the regression analysis the combined HBM values were used as two variables (‘HBM alert’, ‘HBM high’). The status of CMI was available as a binary variable for further analysis.

### Not considered variables, confounder control

An additional source of mercury exposure is fish consumption [4]. The use of a retort during burning amalgam can decrease mercury exposure [2]. For the purpose of this work, the origin of the mercury exposure is not of particular interest [37], thus these factors were not considered.

The symptoms of CMI are similar to those of chronic alcohol intoxication [29, 38]. To avoid a false diagnosis of CMI we tested if alcohol consumption has an influence on the MSS.

### Statistical analysis

The analysis was carried out using the available case analysis, which uses for each analysis all available cases [39]. First, descriptive statistics of the demographic data with medians and frequencies were conducted. Due to nonparametric distribution of all variables except age, the medians and ranges are given, and only non-parametric tests were used. For correlations, the Spearman’s rank correlation coefficient was used. It was tested if age, working with mercury in years and MSS correlate with the VAS and HU values. The Mann-Whitney-U test was used to determine whether the central tendency of the VAS and HU values differ between males and females as well as between participants having CMI and participants not having CMI. For the three categories of the HBM combined limit values the Kruskal-Wallis-test was used.

In order to fulfill the requirements for multiple linear regression analysis, the dependent variables HU and VAS were log transformed. Independent variables were either metric (age, working years, MSS) or binary coded (gender: female ‘0’, male ‘1’; CMI: no ‘0’, yes ‘1’; HBM alert: no ‘0’, yes ‘1’; HBM high: no ‘0’, yes ‘1’).

Four regression models were calculated with VAS and HU as outcomes and two sets of variables. The first set consisted of the variables gender, age, working years, MSS, HBM alert and HBM high. The second set covered of gender, age, working years and status of CMI. Additionally to the exposure variables, gender and age were added for adjustment and for assessing the influence on the outcome. Years of working with mercury was included in the regression analysis for assessing the effect of the duration working with mercury on the HRQoL. For interpretation, the unstandardized coefficients and their 95% confidence interval were back-transformed with the formula (100 ∗ (*e*^*β*^ − 1)). They are presented as change in % in the outcome variable (VAS or HU) if the independent variable (gender, age, etc.) increases by one unit. Analysis were conducted using IBM SPSS Statistics, Version 25.

## Results

### Basic characteristics

In total, 207 persons were included (mean age 38 years, ranging 18 to 77 years, 82% male) as presented in Tables 3 and 4. The median duration of years working with mercury was 10 years ranging from under 1 year up to 48 years. The maximum value of MSS was 8 and the median was 1. Alcohol was found to not be a confounding factor for MSS in this sample (Additional file 8). 67% of the participants had a mercury level below the HBM I (alert) limit value in all specimens, 12% had a mercury level above the HBM II (high) limit value in at least one specimen. In the sample, 9 participants were diagnosed with CMI (Additional file 6). The local medical staff did not notice any chronic diseases. In addition, when participants denied being ‘healthy now’ and were asked why, none of them reported any chronic disease (Additional file 9).

**Table 3:** Basic characteristics (1) and group comparisons

		N (%)	VAS		HU_1_	
			Median (Min.–Max.)	*p*-value	Median (Min.–Max.)	*p*-value
Total sample		207 (100)	80.00 (50–100)		100 (26.90–100)	
Gender	Male	169 (81.6)	81.00 (50–100)	*p* = 0.258_a_	100 (26.90–100)	*p* = 0.095_a_
	Female	38 (18.4)	80.00 (50–100)		83.85 (41.70–100)	
	Missing	0 (0)				
HBM combined	All specimens below HBM I	138 (66.7)	80.50 (50–100)	*p* = 0.169_b_	100 (41.70–100)	*p* = 0.730_b_
	At least one specimen above HBM I	45 (21.7)	86.50 (50–100)		100 (26.90–100)	
	At least one specimen above HBM II	24 (11.6)	80.00 (50–100)		100 (55.20–100)	
	Missing	0 (0)				
CMI	No	197 (95.2)	80 (50–100)	*p* = 0.025*_a_	100 (41.70–100)	*p* = 0.033*_a_
	Yes	9 (4.3)	70 (50–97)		77.70 (26.90–100)	
	Missing	1 (0.5)				

*VAS* visual analogue scale, *HU* health utilities, *HBM combined* human biomonitoring limit values combined from urine and blood mercury levels, *CMI* chronic mercury intoxication, *Min.* Minimum, *Max.* Maximum

1 = two missing values; * = significance level 0.05; ** = significance level 0.01

a = Mann-Whitney-U-test (comparison of central tendency of Outcome between two groups); b = Kruskal-Wallis-Test (comparison of central tendency of Outcome between more than two groups, with adjusted *p*-value)

**Table 4:** Basic characteristics (2) and Spearman’s rank correlations

	N (%)	Median (Min.–Max.)	VAS		HU_1_	
			corr. coeff.	Sig.	corr. coeff.	Sig.
Age	207 (100)	38.00 (18–77)	r_s_ = − 0.186	0.007**	r_s_ = − 0.082	0.242
Working years	207 (100)	10.00 (0.1–48)	r_s_ = − 0.016	0.821	r_s_ = − 0.045	0.526
MSS	204 (98.5)	1.00 (0–8)	r_s_ = − 0.178	0.011*	r_s_ = − 0.210	0.003**
Missing	3 (1.5)					

*VAS* visual analogue scale, *HU* health utilities, *MSS* medical score sum, *Min.* Minimum, *Max.*Maximum, *corr. Coeff.* correlation coefficient, *r*_*s*_ Spearman’s rank correlation coefficient

1 = two missing values; * = significance level 0.05; ** = significance level 0.01

### Health related quality of life (HRQoL)

**Fig. 1:**
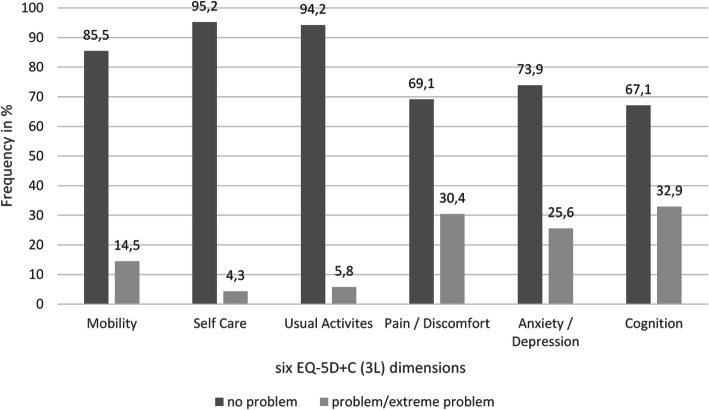
Frequency of reported problems in EQ-5D+C (3L) dimensions

For the VAS, values between 50 and 100 were reported, with a median of 80. The HU values had a median of 100, a minimum of 26.9 and a maximum of 100. For comparison with other studies, means are presented in the Additional file 1. Out of 729 possible EQ-5D + C (3 L) health states, in this sample 40 different health states were reported (Additional file 4). The most common (42.5%) health state was ‘111111’, the worst reported health state was ‘223322’ (0.5%). Most problems were reported in the dimensions cognition (32.9%), pain (30.4%) and anxiety/depression (25.6%) (Fig. 1). All health states and the health states of participants having CMI and are presented in the Additional files 3, 4 and 7).

The VAS and the HU values of participants were not always consistent, e.g. participants, who stated their health with 100 on VAS, had a HU value between 67 and 100, and participants with a HU value of 100 had VAS values between 50 and 100 (Additional file 5).

### Group differences and correlations

The VAS was significantly negatively correlated with age and MSS (rs = − 0.186, *p* = 0.007; − 0.178, *p* = 0.011). This means the higher the age and the higher the MSS, the lower is the VAS value, but the correlations are weak. Participants having CMI had a significant lower VAS (median = 70) compared to not intoxicated participants (median = 80). Furthermore, none of the participants having CMI stated 100 on the VAS. The three HBM groups and gender did not significantly differ regarding VAS values, as well as HU values. Referring to the HU values, there is a significant negative weak correlation with the MSS (rs = − 0.210, *p* = 0.003). Furthermore there is a significant difference in the HU values between the group of participants having CMI (median = 77.7) compared with the participants who did not have CMI (median = 100), as seen in Table 3.

### Regression analysis

All models were significant overall and explained up to 7.5% of the variance in the outcome variable, as seen in Tables 5 and 6.

**Table 5:** Multiple log-linear regression analysis with age, gender, years of working, MSS, HBM alert, HBM high as independent variables

**VAS** **(r**^**2**^** = 0.058)**	**un. coeff.**	**SE**	**95% CI**		***p*-value**	**change in %**	**95% CI**	
Constant	4.470	0.068	4.335	4.604	0.000**	8634.449	7535.439	9891.648
Gender	0.035	0.038	− 0.041	0.111	0.363	3.569	−3.993	11.726
Age	−0.003	0.002	−0.006	0.000	0.042*	−0.327	− 0.642	− 0.012
Years of working	0.004	0.002	0.000	0.009	0.052	0.446	−0.004	0.898
HBM alert	0.042	0.036	−0.029	0.112	0.246	4.249	−2.854	11.872
HBM high	−0.106	0.044	−0.194	−0.019	0.018*	−10.070	−17.612	−1.839
MSS	−0.031	0.012	−0.054	−0.008	0.008**	−3.069	−5.264	−0.823
**HU** **(r**^**2**^** = 0,075)**	**un. coeff.**	**SE**	**95% CI**		*p*-**value**	**change in %**	**95% CI**	
Constant	4.496	0.058	4.382	4.611	0.000**	8868.573	7898.545	9956.242
Gender	0.042	0.033	−0.022	0.106	0.199	4.291	−2.200	11.213
Age	0.000	0.001	−0.002	0.003	0.838	0.028	−0.241	0.297
Years of working	0.001	0.002	−0.002	0.005	0.492	0.133	−0.248	0.515
HBM alert	0.010	0.031	−0.051	0.070	0.749	0.985	−4.930	7.268
HBM high	−0.039	0.038	−0.113	0.036	0.310	−3.778	−10.695	3.676
MSS	−0.044	0.010	−0.063	−0.024	0.000**	−4.275	−6.122	−2.392

*r*^*2*^ adjusted r square; *VAS* visual analogue scale, *HU* health utilities, *HBM alert and HBM high* human biomonitoring limit values combined from urine and blood mercury levels, *MSS* medical score sum, *un. coeff.* unstandardized coefficient, *SE* standard error, *CI* confidence interval

* = significance level 0.05; ** = significance level 0.01

**Table 6:** Multiple log-linear regression analysis with age, gender, years of working, CMI as independent variables

**VAS** **(r**^**2**^** = 0,035)**	**un. coeff.**	**SE**	**95% CI**		*p*-**value**	**change in %**	**95% CI**	
Constant	4.445	0.064	4.318	4.571	0.000**	8416.018	7402.838	9566.016
Gender	0.029	0.038	−0.047	0.104	0.456	2.898	−4.569	10.949
Age	−0.004	0.002	−0.007	−0.001	0.022*	−0.353	−0.654	−0.052
Years of working	0.004	0.002	−0.001	0.008	0.086	0.384	−0.054	0.825
CMI	−0.160	0.073	−0.304	- 0.017	0.029*	−14.823	−26.214	−1.675
**HU** **(r**^**2**^** = 0,054)**	**un. coeff.**	**SE**	**95% CI**		*p*-**value**	**change in %**	**95% CI**	
Constant	4.475	0.054	4.369	4.582	0.000**	8683.679	7797.789	9668.939
Gender	0.030	0.032	−0.033	0.093	0.356	3.005	−3.292	9.711
Age	−0.001	0.001	−0.003	0.002	0.639	−0.060	−0.314	0.194
Years of working	0.001	0.002	−0.002	0.005	0.438	0.144	−0.222	0.512
CMI	−0.227	0.061	−0.347	−0.107	0.000**	−20.283	−29.305	−10.109

*r*^*2*^ adjusted r square, *VAS* visual analogue scale, *HU* health utilities, *CMI* chronic mercury intoxication, *un. coeff.* unstandardized coefficient, *SE* standard error, *CI* confidence interval

* = significance level 0.05; ** = significance level 0.01

The significant determinants of the VAS in the first set of variables (Table 5) were age, HBM high and MSS. Age had a significant negative influence on the VAS value (Change in % = − 0.327; 95% CI [− 0.642; − 0.012]), for every additional year of age, the VAS value decreases by 0.3%. Furthermore, having at least one specimen above the HBM II (high) limit value decreases the VAS value significantly by 10% (Change in % = − 10.070; 95% CI [− 10.070; − 17.612]). A significant 3% decrease in the VAS value derives for each additional symptom (Change in % = − 3.069; 95% CI [− 5.264; − 0.823]). With the first set of variables, the HU is only significantly determined by the MSS score, with a decrease of 4% in the HU value for each additional symptom (Change in % = − 4.275; 95% CI [− 6.122; − 2.392]).

Regressing VAS on the second set of variables (Table 6), age (Change in % = − 0.353; 95% CI [− 0.654; − 0.052]) and the status of CMI (Change in % = − 14.823; 95% CI [− 26.214; − 1.675]) were significant determinants of the VAS. For every additional year of age, the VAS value decreases by 0.4%, having CMI decreases the VAS value by 15%. For HU the status CMI was a significant determinant (Change in % = − 20.283; 95% CI [− 29.305; − 10.109]) and decreases HU values by 20% when having CMI.

## Discussion

To our knowledge this is the first study assessing the HRQoL and analyzing influencing factors among people living in ASGM areas in Zimbabwe. Overall the study sample reported a rather good HRQoL. In summary, it was found that the HRQoL, represented by the VAS and HU values, is significantly determined by the MSS while taking into account age, gender, years of working with mercury and the HBM I and HBM II combined limit values. In addition, the status of CMI had a significant influence on HRQoL while taking into account gender, age and years working with mercury. With every additional symptom caused by a chronic mercury exposure the HRQoL decreases. Having CMI as well decreases the HRQoL. The number of years working with mercury did not show an influence on the HRQoL.

Furthermore, the VAS was determined by the HBM II (high) combined limit value in the first set of variables and by age in both sets of variables. The VAS value decreases with every additional symptom, with having a mercury level in at least one specimens above the HBM II (high) limit value and with every additional year of age. In the second set of variables, the VAS values decrease with having CMI and with every additional year of age.

### Health related quality of life (HRQoL)

The health states as part of the HRQoL of this sample were better than those assessed in previous studies. Becker et al. [21] described that 38.1% of miners in ASGM reported no problem in any dimension. Regarding a sample of the general Zimbabwean population, cognition was not assessed, 47.8% reported no health problem in any dimension [35]. When excluding the dimension cognition, in the sample of this study 54.1% reported no problem in any dimension (Additional file 4). Most problems were reported in the dimension cognition (Fig. 1), corresponding with Becker et al. [21]. Since mercury exposure causes neurological disorders predominantly [4], exposure to mercury of people in ASGM could be the explanation for the large proportion of reported problems in this dimension.

The VAS values with means of 79.8 [35] and 81 [21] of prior studies are consistent to the mean of 80.6 in this study (Additional file 1), though the range of values was wider (35; 100) in Becker et al. [21]. At first, the slightly better HRQoL is surprising. It was assumed that a worse HRQoL would be recorded compared to prior studies due to the aggravating economic crisis [40, 41]. From another point of view, this could be the explanation for the better HRQoL. Mostly ASGM is a poverty driven [16, 17], but at the same time it is a sustainable occupation [14] and earning are higher than in the agricultural sector [26]. Especially with an insecure local currency, gold gains importance as a substitute currency or for securing foreign currencies [1, 23]. Presumably, people living in ASGM areas are still able to earn money with gold trading and therefore state their HRQoL comparably high, despite the circumstances.

Four participants of this study, one having CMI (Additional files 4 and 7) reported a health state, which is the same or worse than the health state for a moderate (121222) form of chronic metallic mercury vapor intoxication assessed by expert interviews [22]. This could mean, that experts focus on the disease and its health limitations and therefore rate a health condition worse than the affected people would rate it [42]. None of the participants stated a health state which corresponds to the health state for a severe (233333) form of chronic metallic mercury vapor intoxication [22]. It is assumable, that people suffering from the most severe form of this intoxication are no longer able to work [15] or to participate in such a study like this.

It can be discussed if the health states of a moderate and severe form of chronic metallic mercury vapor intoxication need adjustment.

### Influencing factors

#### Gender

The gender distribution of this study population does not represent the gender distribution of the Zimbabwean population (47.9% male, 52.1% female) [43] nor the gender distribution in ASGM in Zimbabwe (50% male, 50% female) [13]. In Zimbabwe as well as in the global mean, about half of the people working in ASGM are women, but often they are invisible. They render services to mining areas such as food preparation, sex work, and in mining digging, moving, washing or processing [44]. Further, women are generally discriminated and disadvantaged, while having limited access to resources and land [45]. The discrimination and the invisibility of women in ASGM could be a reason that less women than men could be recruited for this study. The sample population of people who identified themselves as miners and the applied method of snowball sampling may have amplified this effect.

Furthermore, gender does not determine the HRQoL significantly, though women report lower HRQoL in the general population [46] and in mining areas in India [47]. Due to their disadvantaged and discriminated role, we assume, that the objective as well as the subjective HRQoL is worse among the female population in Zimbabwean ASGM areas compared to the male population. Whether there is no gender difference between the HRQoL or if the female proportion of this study sample is too small to detect it, cannot be answered with this study. For men living in areas of ASGM in Zimbabwe this study can make a statement, for women the results are meagre and further assessment is needed.

#### Age

Prior studies found different effects of age. Amponsah-Tawiah et al. found that low and high age can lead to more injuries and the quality of life increases with older age [18]. Jelsma and Ferguson found age to be a negative significant predictor for VAS in a social diverse South African community [48]. In general, the VAS values decrease with age [46]. Consistent with Jelsma and Ferguson [48] and Szende et al. [46], in this study age was found to be a negative predictor for the HRQoL. The negative influence of age on HRQoL in the sample of this study could be explained by two aspects. ASGM involves heavy physical work and is often done by unskilled workers [23], doing this over years or decades can lead to injuries and negatively affects physical health. Furthermore physical limitations hinder from working and therefore hinder from earning money.

#### Years of working with mercury

It is known that a longer duration of working with mercury increases the risk for CMI [1, 5, 6]. Thus, we assumed that the duration of working with mercury also has a negative influence on the HRQoL. In this study, we did not find an effect of the duration working with mercury on the HRQoL while adjusting for age, gender and adverse health conditions caused by exposure to mercury. This might be due to fact that age and the duration of working are similar in content, the years of working are dependent on age. Furthermore, the selection bias in the study sample (see Limitations) could have had an influence on the result.

#### Medical score sum and HBM limit values

The negative correlation of MSS with VAS and HU is seen again in the regression analysis, additional symptoms influence VAS and HU negatively. The HBM combined variable, which represents the mercury exposure in the body, did not show a clear result regarding its the influence on the HRQoL. This is not surprising, because chronic exposure to mercury is asymptomatic for a long time [5, 6]. On the contrary, symptoms like memory loss or tremor are perceptible and therefore the influence on how someone feels is much more direct. Having CMI influences the HRQoL in a negative way. This finding implies, that symptoms seem to be a very important factor that influences the HRQoL. Additionally this shows how dangerous the exposure to mercury is for human health. Measuring the HRQoL could work as alert system, however, just if exposure already results in symptoms, which reduce the HRQoL.

Besides that, our finding supports why a diagnosis of CMI should be done with a combination of symptoms and mercury exposure values in the body [28].

#### Chronic mercury intoxication

Compared to other studies [5, 24] the rate of CMI is with only 4.3% very low. The mean weighted prevalence over pooled studies for CMI is 23.7% among population working or being at the mining site [15]. Among active miners and gold shop workers, the mean weighted prevalence is even higher with 34.3% [15]. Mambrey et al. assessed exposure risk factors for CMI among people who identified themselves as miners in Zimbabwe [49].

This sample consists of people living in ASGM areas, not only of people working and being at the mining site. This might explain the difference between the prevalence rates of CMI. The possibility that working conditions changed, and the mercury exposure diminished was denied by the local project partner from Baines Occupational and Travel Medicine Centre and Midlands State University (Zimbabwe).

#### Methodological considerations

The results of the regression analysis show a clear trend of the same variables having an influence on the VAS and HU values, despite the following differences between the VAS and HU values. The HU are calculated from the health states using population-based weights. Due to the fact, that there is no weight available for the dimension cognition, the HU are calculated with only five dimensions, whereas cognition is included in rating the VAS value. Furthermore, the VAS and HU values are not always consistent within participants. It means, the participant rated his/her health state differently than the general population would rate the same health state. This inconsistency shows that VAS and HU seem to measure different aspects concerning the HRQoL. The differences between the outcomes on one hand and the same trend in the results on the other hand, support the decision to use VAS and HU as outcomes to describe the HRQoL.

Regarding the CMI, Doering et al. concluded it could be checked if mercury limit values need to be adjusted [28], which would entail a change in the diagnosis of CMI.

There are different instruments to assess the HRQoL. The decision on the instrument depends on the purpose and the study design [50]. Among diamond miners, there were significant differences between the used instruments [51]. Amposah-Tawiah et al. nor Noronha and Nairy used the EQ-5D as instruments for assessing HRQoL [18, 47].

The EQ-5D + C (3 L) is user friendly, easy to understand [31] and available in English and further languages like Shona [52]. The target population often has low literacy [17], hence the comprehensibility and the availability in Shona are big advantages. Those advantages and the international comparability [53] were the reasons why the EQ-5D + C (3 L) was chosen to assess the HRQoL. On the other hand a ceiling effect was seen concerning the EQ-5D [54, 55], a bigger proportion of study participants reported full health on the EQ-5D compared to a different instrument assessing the HRQoL (SF-6D). This effect might apply in this study as well, but without a comparison instrument this cannot be tested.

The multiple log-linear regression analysis explained up to 7.5% of the variance in the outcome variables, this means the majority of influencing factors were not assessed. Jelsma and Ferguson found the perceived socioeconomic standing to have an influence on the HRQoL [48]. Furthermore, among different patient groups the following aspects were assessed which are not captured by the EQ-5D: work limitations, social life, family relationships, financial issues, received medical and social care [56]. Objective conditions in mining areas like the access to resources influence the living conditions [47], and presumably also the HRQoL. For ASGM areas in Zimbabwe those issues are quite possible. The access to land is not well managed and therefore there is conflict and fight [14] persisting due to informality of ASGM [17]. A higher socioeconomic standing with better social support and better access to resources could reduce those barriers.

On the other hand, previous studies assessed the socio-economic status of gold miners in Zimbabwe [23, 26], the average income is between 10 to 40 US dollars per month [21, 23] and is higher than the average income in the agricultural sector [26]. People working in mining earn more money and save more money than those farming [26], that could explain the high HRQoL in the sample of this study. Becker et al. assessed the HRQoL of gold miners and compared the findings with the general Zimbabwean population, they did not find income to be an influencing factor on HRQoL [21].

### Limitations

A source for uncertainty is the method of recruitment and the place of conducting the study as well as the nonprobability sampling. We did not calculate the sample size as we would like to have an explorative case study to see the general picture about the HRQoL and the influence of mercury exposure among people working in ASGM in Zimbabwe. We, therefore, adopted a nonprobability sampling method to include only volunteers in our study. The initial intention was to reach a population actually working in the ASGM sector. Furthermore, we planned to stratify the sample according to their job. Both information was assessed by asking a question concerning their type of job. Due to several reasons, we cannot be sure whether participants answered the question concerning their job honestly. First, the data collection was conducted at health centers and not at the mining site. This was necessary to avoid contamination of the specimens, albeit this complicates accessibility to the target population and leads to less control who participates in the study. Furthermore, due to the tense economic situation in the country at that time [40, 57] we assume that some people participated in order to receive the 5 US dollars, although they do not work as gold miners as their main job. Therefore, the variable was not considered in the analysis and the influence of the type of job on the HRQoL cannot be answered. This resulted in a study population of people who identified themselves as miners and live in ASGM areas in Zimbabwe. Nevertheless, we could model the relation between adverse health conditions caused by chronic exposure to mercury and the HRQoL.

Furthermore, there is another selection bias in the sample. Although the study does not compare workers with the general population, the healthy-workers bias applies to the study sample [58]. In ASGM, minor, but also major and deadly accidents occur [59], disabled people or very severe cases of CMI were not among the participants and therefore are not recorded as seen before by Drasch et al. [29]. This leads towards a healthier sample than the target population assembly is.

The results are only limitedly transferable to other countries, because mining conditions can differ even within a country [23]. This is one reason why there is not one fitting solution for solving problems in ASGM.

### Recommendations

Further research should seek recruit a larger population sampled by probability methods and to assess reliable information on the occupational status of the participants. For a better understanding which factor influence the HRQoL among people living and working in ASGM areas, additional variables like socioeconomic status and objective conditions should be collected. The HRQoL could be assessed using different instruments for evaluating the most appropriate instrument. Also a disease specific instrument would be conceivable [47]. A disease specific instrument could also be applied in combination with the EQ-5D questionnaire [53]. The high number of reported problems in the dimension cognition could be investigated further and whether it is attributable to the occupational mercury exposure.

Further studies should make an effort to reach approximately as many women as men for the study sample in order to assess possible differences, which need to be taken into account when planning interventions. We advise further, to assess especially vulnerable and invisible groups affected by chronic exposure to mercury, like children [23] and people who are former miners and stopped working due to bad health condition [29].

As almost everyone in Zimbabwe has a cell phone, one idea for an intervention could use text messages and a snowball system to disseminate information to fill knowledge gaps [23], this would be cheap and in comparison easy to implement.

## Conclusion

This study shows, that adverse health effects caused by chronic exposure to mercury, have a negative influence on the HRQoL among people living in ASGM areas. Especially neurological symptoms caused by exposure to mercury seem to have a decisive influence on the HRQoL. Despite the limitations, this study illustrates some of the challenges and hazardous impacts that people in ASGM areas face. A holistic understanding is fundamental planning and implementing interventions targeting working and living conditions in ASGM areas, improving HRQoL of people and implementing the ‘Minamata Convention on Mercury’.

## Supplementary information

**Additional file 1.** Means, medians, standard deviation, range, minimum and maximum of VAS and HU.

**Additional file 2.** Eleven most frequent health states.

**Additional file 3.** Frequency and percentage of 11 most frequent health states.

**Additional file 4.** Frequency and percentage of all health states.

**Additional file 5.** Scatterplot Visual analogue scale and health utilities.

**Additional file 6.** Assessment of chronic mercury intoxication (CMI) in the study sample.

**Additional file 7.** Health states of participants with chronic mercury intoxication.

**Additional file 8.** Alcohol consumption with Medical Score Sum (MSS).

**Additional file 9.** Clustered answers to the question "are you healthy now" if negated.

## Data Availability

The datasets used and/or analyzed during the current study are available from the corresponding author on reasonable request.

## Abbreviations

ASGM: Artisanal and small-scale gold mining
CMI: Chronic mercury intoxication
EQ-5D + C (3 L): The five EuroQol (EQ) dimensions (5D: mobility, self-care, usual activities, pain/discomfort, anxiety/depression) plus cognition (EQ-5D + C) questionnaire assessed with three levels (3 L: no, some, extreme problems)
HRQoL: Health-related quality of life
HBM: Human Biomonitoring
HU: Health utility
MSS: Medical score sum
VAS: Visual analogue scale
UNEP: United Nations Environmental Program
US: United States
