# Supplementary material for: Health-related quality of life (EQ-5D + C) among people living in artisanal and small-scale gold mining areas in Zimbabwe: a cross-sectional study
Source: Health Qual Life Outcomes. 2020 Aug 18;18:284. doi: 10.1186/s12955-020-01530-w (PMC7437047; doi:10.1186/s12955-020-01530-w)
Supplement: Supplementary file 1 — Additional file 1. Means, medians, standard deviation, range, minimum and maximum of VAS and HU. [file 12955_2020_1530_MOESM1_ESM.docx]

Additional File 1: Means, medians, standard deviation, range, minimum and maximum of VAS and HU

|  | N | Missing | Mean | Median | Standard deviation | Range | Minimum | Maximum |
| --- | --- | --- | --- | --- | --- | --- | --- | --- |
| Visual Analog Scale (VAS) | 207 | 0 | 80.560 | 80 | 15.741 | 50 | 50 | 100 |
| Health utility (HU) | 205 | 2 | 89.596 | 100 | 13.368 | 73.100 | 26.900 | 100 |
