# Supplementary figures and images for: Health-related quality of life (EQ-5D + C) among people living in artisanal and small-scale gold mining areas in Zimbabwe: a cross-sectional study
Source: Health Qual Life Outcomes. 2020 Aug 18;18:284. doi: 10.1186/s12955-020-01530-w (PMC7437047; doi:10.1186/s12955-020-01530-w)

Additional File 2: Eleven most frequent health states

**
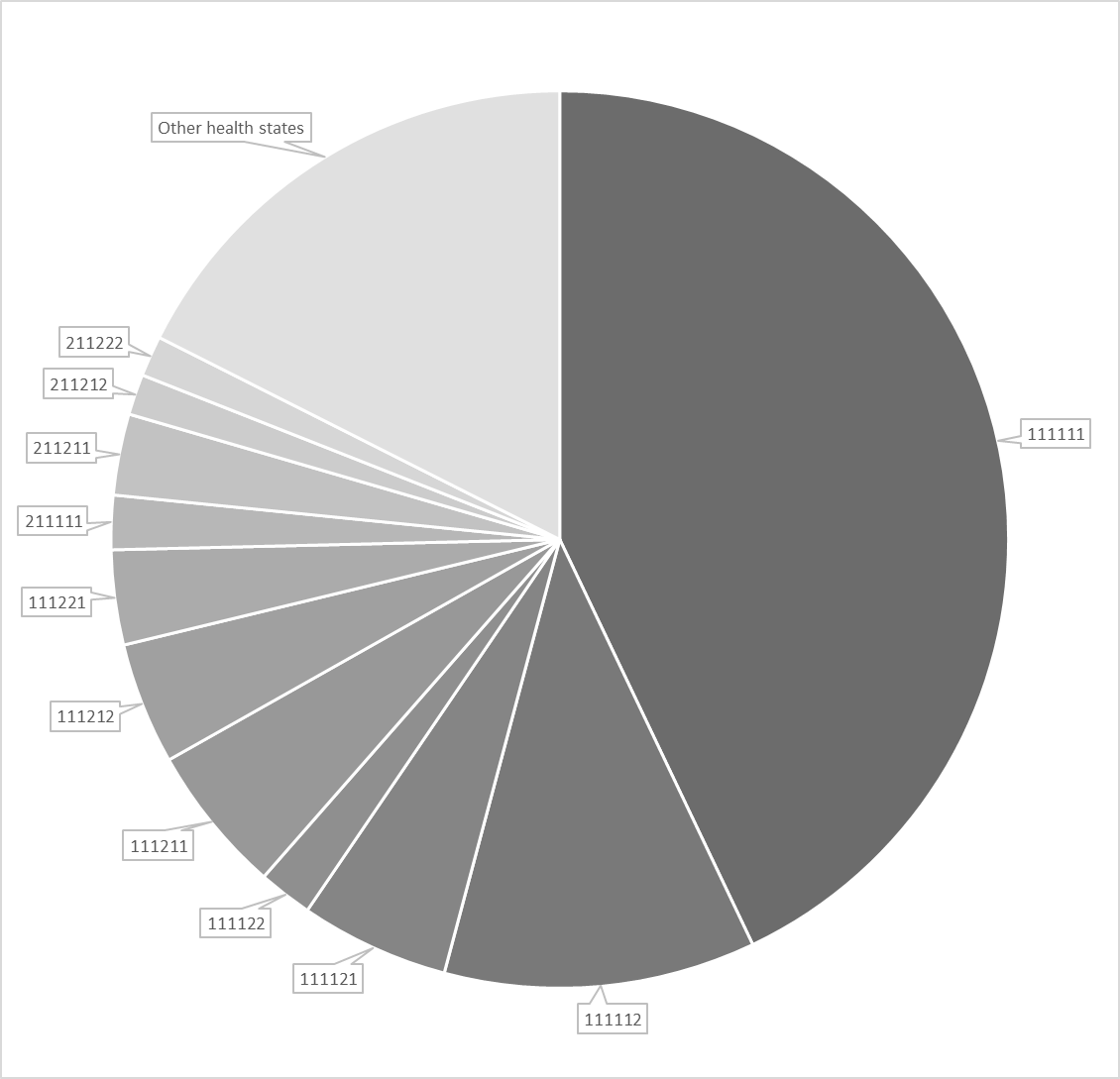
**

Supplement: Supplementary file 2 — Additional file 2. Eleven most frequent health states. [file 12955_2020_1530_MOESM2_ESM.docx]

Additional File 5: Scatterplot Visual analogue scale and health utilities


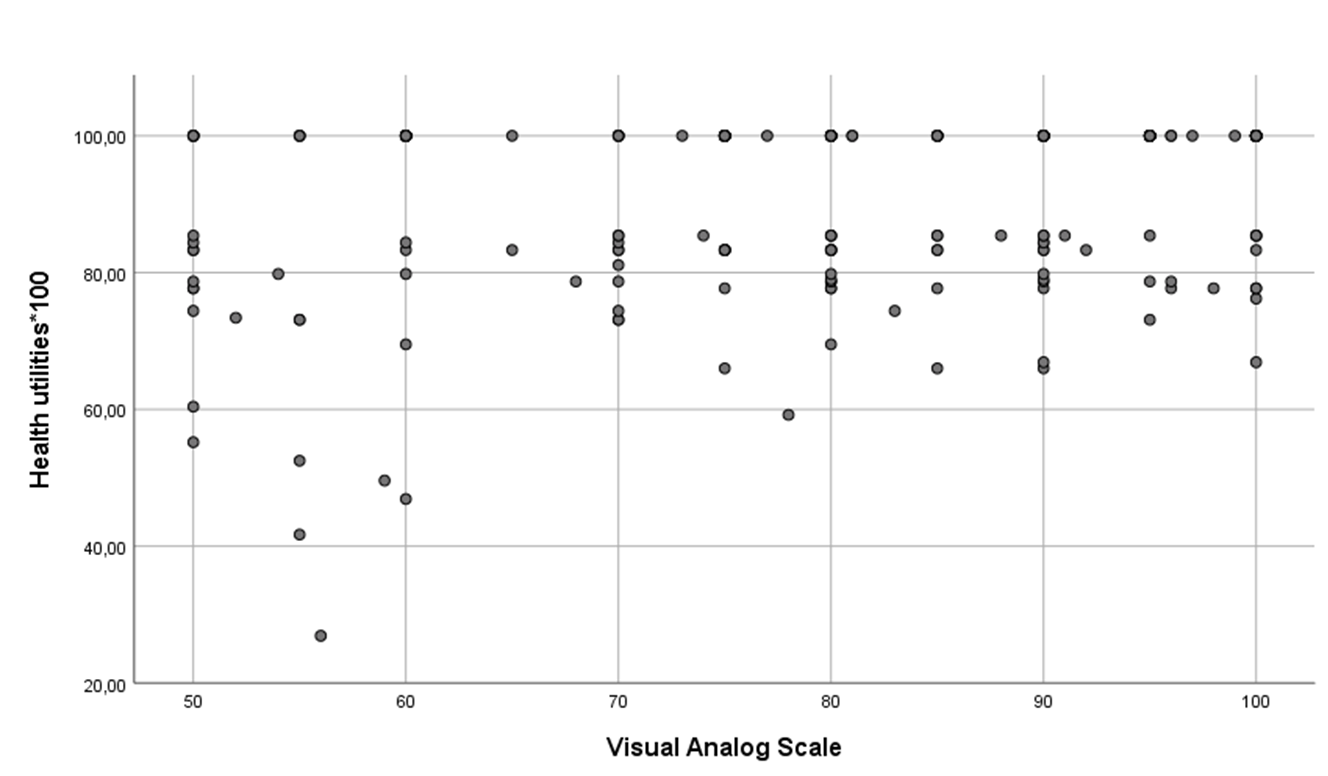

Supplement: Supplementary file 5 — Additional file 5. Scatterplot Visual analogue scale and health utilities. [file 12955_2020_1530_MOESM5_ESM.docx]
