# Supplementary material for: Health-related quality of life (EQ-5D + C) among people living in artisanal and small-scale gold mining areas in Zimbabwe: a cross-sectional study
Source: Health Qual Life Outcomes. 2020 Aug 18;18:284. doi: 10.1186/s12955-020-01530-w (PMC7437047; doi:10.1186/s12955-020-01530-w)
Supplement: Supplementary file 3 — Additional file 3. Frequency and percentage of 11 most frequent health states. [file 12955_2020_1530_MOESM3_ESM.docx]

Additional File 3: Frequency and percentage of 11 most frequent health states

| Health state | Frequency | Valid % |
| --- | --- | --- |
| 111111 | 88 | 42,9 |
| 111112 | 23 | 11,2 |
| 111121 | 11 | 5,4 |
| 111122 | 4 | 2,0 |
| 111211 | 11 | 5,4 |
| 111212 | 9 | 4,4 |
| 111221 | 7 | 3,4 |
| 211111 | 4 | 2,0 |
| 211211 | 6 | 2,9 |
| 211212 | 3 | 1,5 |
| 211222 | 3 | 1,5 |
| Other health states | 36 | 17,6 |
| sum | 205 | 100,0 |
| missing | 2 |  |
| total | 207 |  |
