# Supplementary material for: Health-related quality of life (EQ-5D + C) among people living in artisanal and small-scale gold mining areas in Zimbabwe: a cross-sectional study
Source: Health Qual Life Outcomes. 2020 Aug 18;18:284. doi: 10.1186/s12955-020-01530-w (PMC7437047; doi:10.1186/s12955-020-01530-w)
Supplement: Supplementary file 4 — Additional file 4. Frequency and percentage of all health states. [file 12955_2020_1530_MOESM4_ESM.docx]

Additional File 4: Frequency and percentage of all health states

| Health state | Frequency | Valid % |
| --- | --- | --- |
| 111111 | 88 | 42.9 |
| 111112 | 23 | 11.2 |
| 111121 | 11 | 5.4 |
| 111122 | 4 | 2.0 |
| 111123 | 2 | 1.0 |
| 111211 | 11 | 5.4 |
| 111212 | 9 | 4.4 |
| 111213 | 1 | .5 |
| 111221 | 7 | 3.4 |
| 111222 | 1 | .5 |
| 111231 | 2 | 1.0 |
| 111232 | 1 | .5 |
| 111322 | 1 | .5 |
| 112122 | 1 | .5 |
| 112211 | 1 | .5 |
| 112212 | 1 | .5 |
| 112221 | 1 | .5 |
| 112222 | 2 | 1.0 |
| 113131 | 1 | .5 |
| 121122 | 1 | .5 |
| 121221 | 2 | 1.0 |
| 122232 | 1 | .5 |
| 122323 | 1 | .5 |
| 131111 | 1 | .5 |
| 131112 | 1 | .5 |
| 211111 | 4 | 2.0 |
| 211112 | 1 | .5 |
| 211121 | 1 | .5 |
| 211122 | 2 | 1.0 |
| 211123 | 1 | .5 |
| 211211 | 6 | 2.9 |
| 211212 | 3 | 1.5 |
| 211213 | 2 | 1.0 |
| 211221 | 2 | 1.0 |
| 211222 | 3 | 1.5 |
| 211232 | 1 | .5 |
| 211322 | 1 | .5 |
| 212212 | 1 | .5 |
| 222232 | 1 | .5 |
| 223322 | 1 | .5 |
| total | 205 | 100.0 |
| Missing | 2 |  |
|  | 207 |  |

Colored cells are health states corresponding
to the health states for a moderate form of
chronic metallic mercury vapor
intoxication assessed by experts (22)
