# Supplementary material for: Health-related quality of life (EQ-5D + C) among people living in artisanal and small-scale gold mining areas in Zimbabwe: a cross-sectional study
Source: Health Qual Life Outcomes. 2020 Aug 18;18:284. doi: 10.1186/s12955-020-01530-w (PMC7437047; doi:10.1186/s12955-020-01530-w)
Supplement: Supplementary file 6 — Additional file 6. Assessment of chronic mercury intoxication (CMI) in the study sample. [file 12955_2020_1530_MOESM6_ESM.docx]

Additional File 6: Assessment of chronic mercury intoxication (CMI) in the study sample

|  |  | MSS  0-2 | MSS  3-4 | MSS 5-10 | Missing | total |
| --- | --- | --- | --- | --- | --- | --- |
| HBM combined | Mercury in both specimens below HBM I | 88 | 23 | 1 | 0 | 112 |
|  | Mercury at least in one specimen between HBM I and HBM II | 36 | 11 | 2 | 1 | 50 |
|  | Mercury at least in one specimen above HBM II | 36 | 5 | 2 | 0 | 43 |
|  | missing | 2 | 0 | 0 | 0 | 2 |
|  | total | 162 | 39 | 5 | 1 | 207 |

Colored cells are considered as chronic mercury intoxicated,

HBM I = 1^st^ exposure limit value, HBM II = 2^nd^ exposure limit value, MSS = Medical score sum
