# Supplementary material for: Health-related quality of life (EQ-5D + C) among people living in artisanal and small-scale gold mining areas in Zimbabwe: a cross-sectional study
Source: Health Qual Life Outcomes. 2020 Aug 18;18:284. doi: 10.1186/s12955-020-01530-w (PMC7437047; doi:10.1186/s12955-020-01530-w)
Supplement: Supplementary file 7 — Additional file 7. Health states of participants with chronic mercury intoxication. [file 12955_2020_1530_MOESM7_ESM.docx]

Additional File 7: Health states of participants with chronic mercury intoxication

| Health state | Frequency of chronic mercury intoxication |
| --- | --- |
| 111111 | 2 |
| 111112 | 1 |
| 111211 | 1 |
| 113131 | 1 |
| 211211 | 2 |
| 211232 | 1 |
| 223322 | 1 |
| total | 9 |

Colored cells are health states corresponding
to the health states for a moderate form of chronic metallic

mercury vapor intoxication assessed by experts (22)
