# Supplementary material for: Health-related quality of life (EQ-5D + C) among people living in artisanal and small-scale gold mining areas in Zimbabwe: a cross-sectional study
Source: Health Qual Life Outcomes. 2020 Aug 18;18:284. doi: 10.1186/s12955-020-01530-w (PMC7437047; doi:10.1186/s12955-020-01530-w)
Supplement: Supplementary file 8 — Additional file 8. Alcohol consumption with Medical Score Sum (MSS). [file 12955_2020_1530_MOESM8_ESM.docx]

Additional File 8: Alcohol consumption with Medical Score Sum (MSS)

| Alcohol consumption | N | MSS Mean (min.-Max. | p-value |
| --- | --- | --- | --- |
| Never | 71 | 1 (0–5) | 0.27 |
| At least once a month | 15 | 1 (0–3) |  |
| At least once a week | 45 | 1 (0–5) |  |
| At least once a day | 45 | 1 (0–3) |  |
| Gesamt | 176 | 1 (0–5) |  |

MSS = Medical score sum; b = Kruskal-Wallis-Test (comparison of central tendency of Outcome between more than two groups, with adjusted p-value)
