# Supplementary material for: Health-related quality of life (EQ-5D + C) among people living in artisanal and small-scale gold mining areas in Zimbabwe: a cross-sectional study
Source: Health Qual Life Outcomes. 2020 Aug 18;18:284. doi: 10.1186/s12955-020-01530-w (PMC7437047; doi:10.1186/s12955-020-01530-w)
Supplement: Supplementary file 9 — Additional file 9. Clustered answers to the question "are you healthy now" if negated. [file 12955_2020_1530_MOESM9_ESM.docx]

Additional File 9: Clustered answers to the question "are you healthy now" if negated

25.1% (N=52) of the participants state, that they are not healthy at the moment. Free answers were clustered into groups of main symptoms. Of those 52 more than half (55.8%) suffer from pain. The other most common health problems were body weakness (19.2 %), problems with breathing (11.5%), problems with the eyes (13.5%), dizziness (7.7%) and 25% other health problems.
